# Surgical Treatment Options for Renal Cell Carcinoma Metastases to the Pancreas—25 Years of Single-Center Experience

**DOI:** 10.3390/cancers18010004

**Published:** 2025-12-19

**Authors:** Magdalena Gajda, Ewa Grudzińska, Paweł Szmigiel, Paweł Sasiński, Sławomir Mrowiec

**Affiliations:** Department of Gastrointestinal Surgery, Medical University of Silesia, 40-752 Katowice, Poland; ewa.grudzinska@sum.edu.pl (E.G.); pawel256686@wp.pl (P.S.); sasinski.pawel93@gmail.com (P.S.); smrowiec@sum.edu.pl (S.M.)

**Keywords:** renal clear carcinoma, RCC, pancreatic metastases, metastasectomy, metastatic cancer

## Abstract

**Background**: Clear cell renal cell carcinoma (RCC) is the most common primary tumor that metastasizes to the pancreas, and surgery is the established treatment option. The aim of this study was to compare surgical treatment options for RCC metastases to the pancreas and to assess long-term outcomes, identifying risk factors for recurrence and death. **Methods**: We retrospectively analyzed data from 62 patients with RCC metastases to the pancreas who underwent pancreatic surgery at the Department of Gastrointestinal Surgery, Medical University of Silesia, Katowice. Patients were divided into two groups: those who underwent local tumor removal (group A, N = 10) and those who underwent classical pancreatic resection (group B, N = 52). Demographic data, postoperative course, histological findings, and clinical outcomes—recurrence-free survival (PFS) and overall survival (OS)—were analyzed. **Results**: In group A, tumors were smaller (*p* < 0.001) and exclusively single (*p* = 0.100), and Clavien–Dindo complications were milder, with a predominance of grade 0 (90% vs. 28.8%; *p* = 0.042). In group B, blood loss was greater (*p* < 0.001), and hospitalization was longer (median 12.5 days vs. 10.5 days; *p* = 0.022) compared with group A. Group A had a longer PFS (144 months vs. 61 months; *p* = 0.007) and longer OS (144 months vs. 70 months; *p* = 0.006) compared with group B. In the entire cohort, independent factors associated with worse OS in multivariate analysis were larger tumor size (*p* = 0.003), lymphatic invasion (*p* < 0.001), vascular invasion (*p* < 0.001), perineural invasion (*p* < 0.001), R1 resection (*p* < 0.001), and symptoms of the metastases (*p* < 0.001). **Conclusions**: The prognosis following surgical resection of pancreatic RCC metastases is excellent: median OS is 77 months, and 5-year survival reaches 71.4%. In multivariate analysis, the type of surgical treatment is not significantly associated with OS or PFS. The choice of surgical procedure should depend on the preoperative CT results and the intraoperative assessment of the surrounding tissues.

## Simple Summary

Clear cell renal cell carcinoma (RCC) is the most common primary tumor that metastasizes to the pancreas, and surgery is the established treatment option. The aim of this study was to compare surgical treatment options for RCC metastases to the pancreas—local removal of the metastatic tumor while sparing the pancreatic parenchyma with classical surgical resection—and to assess long-term outcomes, identifying risk factors for recurrence and death. According to the results of our study, the prognosis following surgical resection of pancreatic RCC metastases is excellent: median OS is 77 months, and 5-year survival reaches 71.4%. In multivariate analysis, the type of surgical treatment is not significantly associated with OS or PFS. The choice of surgical procedure should depend on the preoperative CT results and the intraoperative assessment of the surrounding tissues.

## 1. Introduction

Renal cell carcinoma (RCC) is the most common type of kidney cancer, accounting for approximately 85% of cases [1]. The incidence of RCC is increasing worldwide, currently estimated at 400,000 new cases per year, and the global mortality rate is nearly 175,000 deaths per year [2]. The overall 5-year survival rate for RCC is approximately 75% but varies significantly depending on the stage of the tumor—for localized disease, the rate is over 90%, while for advanced, metastatic disease, it drops to 20–30% [3]. Nearly 30% of patients with RCC ultimately develop metastases, which typically spread to the lungs, bones, brain, adrenal glands, liver, contralateral kidney, gallbladder, lymph nodes, and pancreas [4,5]. Metastases from RCC to the pancreas are extremely rare, accounting for only 2–5% of all pancreatic malignancies [6]. However, the pancreas is a specific site of RCC metastasis—metastases from RCC are the most frequently reported secondary pancreatic malignancies [7], surpassing metastases from lung cancer, breast cancer, colorectal cancer, and melanoma in incidence [7,8]. Metastatic RCC can be treated with adjuvant therapy, systemic therapy, and surgery [5]. For pancreatic RCC metastases, metastasectomy is a recognized surgical treatment option, with favorable overall survival outcomes—5-year survival rates of 72–88% [9,10,11,12].

However, because RCC metastases to the pancreas are relatively rare, reports in the literature are based primarily on individual reports and small case series, which may make it difficult to draw consistent conclusions. Given the extensive experience of our clinical center, this series of 62 cases of surgical treatment of RCC metastases to the pancreas can contribute to drawing conclusions about the outcomes of surgical treatment of RCC metastases to the pancreas and assessing the oncological consequences of specific surgical procedures.

The aim of the study is to compare the types of surgical treatment used for RCC metastases to the pancreas and to evaluate the results of this treatment (survival after 1, 3, and 5 years), identifying the risk factors for recurrence and death.

## 2. Materials and Methods

### 2.1. Characteristics of the Study Group

**Figure 1:**
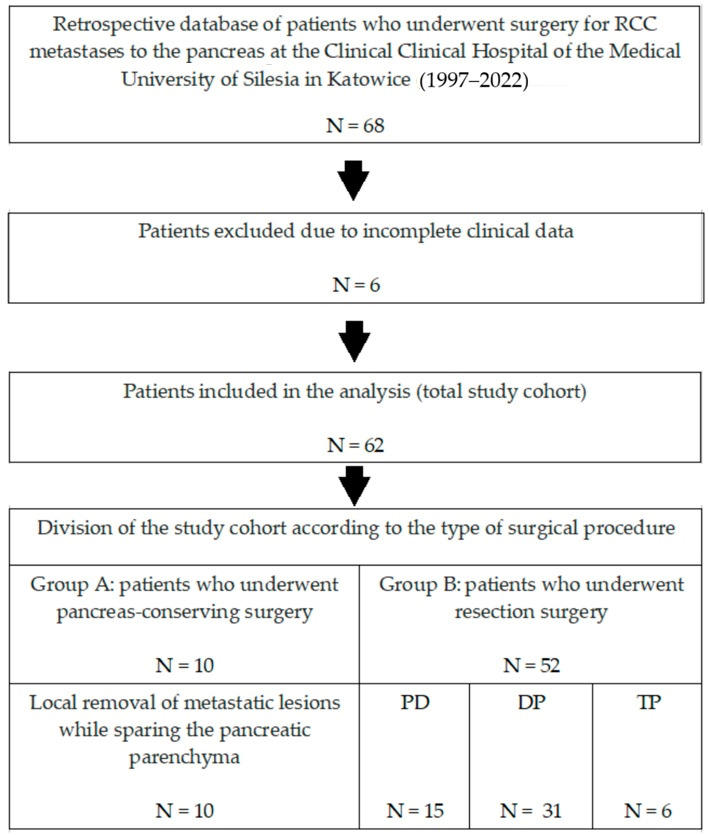
Flowchart showing the screening performed to select patients for the study.

A retrospective analysis of 68 patients with metastatic renal cancer to the pancreas who underwent pancreatic surgery at the Department of Gastrointestinal Surgery of the Medical University of Silesia in Katowice (Poland) between 1997 and 2022 was performed. After excluding 6 patients with incomplete clinical data, data from 62 patients who underwent surgery during the study period were retrospectively analyzed. Demographic data, details of metastatic lesions, information about surgical procedures, analysis of the postoperative course, histological data of postoperative samples, and information on oncological follow-up after surgical treatment were collected. Patients were divided into two groups (Figure 1):

- Group A (N = 10)—patients who underwent limited surgery: removal of only the metastatic tumor while sparing the pancreatic parenchyma.
- Group B (N = 52)—patients who underwent resection: removal of the metastatic tumor with part of the pancreatic parenchyma (pancreaticoduodenectomy—PD or distal pancreatectomy—DP) or the entire pancreatic parenchyma (total pancreatectomy—TP).

Qualification for surgery was based on the results of imaging studies: computed tomography (CT) and/or magnetic resonance (MR) scans of the abdomen and pelvis. Patients with resectable tumor(s), with no imaging evidence of invasion of significant venous vessels (portal vein, superior mesenteric vein) and/or arterial vessels (celiac trunk, common hepatic artery, superior mesenteric artery), were eligible for surgery. Additionally, 30 patients (48.4%) underwent endoscopic ultrasonography (EUS) with biopsy, confirming the diagnosis.

Due to the fact that there is no official expert opinion on the recommended extent of surgery in the case of RCC metastases to the pancreas, the type of surgical procedure (local removal vs. resection) was selected individually by the operator performing the procedure, based on the results of imaging diagnostics and the intraoperative findings. Local removal of the metastatic lesion was applied in cases where the following criteria were simultaneously met:

- The distance of the metastatic lesion from the main pancreatic duct was >3 mm, as determined based on contrast-enhanced CT, analyzed by two experienced radiologists.
- Intraoperative confirmation of a clear delineation of the metastatic lesion from the pancreatic parenchyma and/or the presence of a capsule, without features of parenchymal infiltration.
- Intraoperative, technical possibility of preserving at least a 1 mm margin of macroscopically unchanged pancreatic tissue around the tumor.
- No signs of tumor aggressiveness—lack of tumor infiltration into the extrapancreatic tissues.

Meeting the above criteria allowed for safe, local removal of the metastatic lesion with preservation of the margin and helped to avoid postoperative complications such as pancreatic fistula. In cases where the above criteria were not met, the metastatic lesion was removed through a resection procedure.

Postoperative follow-up was overseen by the operating surgeon or by an oncologist. Follow-up was performed with CT scans of the abdomen and pelvis at 6-month intervals for the first 2 years, followed by 12-month intervals for the next 3 years and 12-month or 24-month intervals for the rest of the patient’s life. CT scan results were analyzed and discussed by the radiologist and surgeon to quickly detect any local recurrence and/or distant metastases.

Our work is a retrospective analysis based solely on the analysis of existing, fully anonymized data, with no patient involvement. As such, in accordance with the national legislation, it is not considered a medical experiment, and the consent of the bioethics committee and the patient’s consent are not required.

### 2.2. Statistical Tests

The level of statistical significance was set at *p* < 0.05. Continuous variables were described using the median, interquartile range (IQR), and range [min, max]. The distribution of categorical variables was presented using the frequency count and percentage for each category. Comparisons between two independent groups of continuous variables were performed using the Wilcoxon rank-sum test. Comparisons between groups of categorical variables were performed using the Pearson chi-square test for variables with sufficient expected frequency (5 or more) and the Fisher exact test for variables with low frequency (<5). For multiple comparisons, the Holm–Bonferroni correction method was used to control type I error. Overall survival (OS), defined as the time from surgery to death from any cause, and progression-free survival (PFS), defined as the time from surgery to local recurrence or distant metastasis (with separate analyses for local recurrence and distant metastasis), were estimated using adjusted Kaplan–Meier curves derived from a stratified Cox proportional hazards model. This model incorporated tumor size as a covariate and propensity score weighting (PSW) to address residual imbalances, allowing for group-specific baseline hazards while adjusting for confounding (Supplementary Materials Tables S1–S7). Greenwood’s standard variance was employed to compute 95% confidence intervals. Differences in OS and PFS between the adjusted Kaplan–Meier curves were evaluated using the Wald test from a companion non-stratified Cox model, which included surgical procedure as a predictor alongside the tumor size covariate, ensuring conditional assessment of group effects under the proportional hazards assumption. Univariate and multivariate analysis of the influence of demographic and clinical characteristics on OS and PFS was performed using a Cox proportional hazards regression model; in the multivariate model, effects were adjusted for age, sex, and body mass index (BMI). The proportional hazards assumption was verified using the Schoenfeld test. Effect sizes were estimated using hazard ratios (HRs). *p*-values were approximated using the Wald test.

### 2.3. Statistical Environment Used

Analyses were performed using the R statistical language (version 4.3.3) on Windows 11 Pro 64-bit (build 26100), using the report (version 0.5.8), ggsurvfit (version 1.1.0), gtsummary (version 2.2.0), survival (version 3.7.0), ggplot2 (version 3.5.0), and dplyr (version 1.1.4) packages.

## 3. Results

### 3.1. Oncological Results

A total of 62 patients with RCC metastases to the pancreas, who underwent surgical treatment at a single center between 1997 and 2022, were analyzed. The retrospective analysis focused on the evaluation of treatment outcomes, taking into account risk factors for recurrence and death, and on the comparison of local removal (group A) and resection (group B) approaches (Table 1).

### 3.2. Analysis of the Entire Study Cohort

The cohort was characterized by a male predominance (66.1%), consistent with the epidemiology of RCC, where the male-to-female ratio is approximately 2:1 [1,2]. The median age of 64.0 years (IQR: 59.0–68.0) indicates a middle-aged to elderly population. The median BMI was 26.4 kg/m^2^ (IQR: 23.7–29.0), and a history of smoking was present in 61.3% of patients. ASA classification showed that 51.6% of patients were in class II (moderate risk), and 32.3% were in class III (high risk), reflecting the moderate burden of comorbidities.

The primary tumor (RCC) was more frequently located in the left kidney (59.7%) than in the right kidney (45.2%). Pancreatic metastases were found to be clear cell renal cell carcinoma (ccRCC) in 100% of cases, which is the most common subtype of RCC (85% of cases) [1]. Pancreatic metastases were located in the head (35.5%), body (41.9%), tail (41.9%), or multifocally (16.1%). Most tumors were single (75.8%), and multiple tumors occurred in 24.2% of patients. The median size of the metastatic tumor was 2.0 cm (IQR: 1.4–2.5). The predominant histological stage was G2 (58.1%), with a smaller proportion of G1 (22.6%) and G3 (19.4%), which implies a predominance of intermediate-grade tumors. Histologically, lymphatic invasion was observed in 21.0% of patients, vascular invasion in 35.5%, and perineural invasion in 21.0%. Metachronous metastases predominated (98.4%), with a median time from nephrectomy of 101.5 months (approximately 8.5 years), suggesting slowly progressive disease. Imaging included CT in 91.9%, MRI in 75.8%, and EUS in 48.4% of patients.

In the treatment of the primary tumor, total nephrectomy predominated (74.2%), with partial nephrectomy performed in 27.4% of patients. In the treatment of metastatic lesions, DP predominated (50.0%), PD was performed in 24.2% of patients, local resection in 16.1% of patients, and TP in 9.7%. R0 margins were achieved in 72.6% of patients. Median blood loss was 300 mL. Clavien–Dindo complications were mostly low-grade (grades 0–II in approximately 71% of patients), and higher grades (III–V in 29% of patients) included reoperations in 11.3% and perioperative deaths in 4.8% of patients. Median hospitalization was 12 days.

Median follow-up for the entire cohort was 77.0 months. Local recurrence occurred in 1.3% of patients and distant recurrence in 29.0%. Median PFS was 72.0 months (6 years), and OS was 77.0 months (approximately 6.4 years), with death occurring in 53.2% of patients.

### 3.3. Analysis of Group A

Group A—patients undergoing local removal of metastatic tumors—consisted of 10 patients, with a male predominance (70%). The median age was 64.0 years (IQR: 59.0–68.0; range: 48.0–71.0), which was similar to the overall cohort. All patients in this subgroup had a history of smoking (100%), and the median BMI was 25.5 kg/m^2^ (IQR: 23.7–30.4; range: 21.8–31.9). ASA classification was predominant in class II (70%), with class III in 30% and no class I. Preoperative diagnostic workup included CT in 80%, MRI in 80%, and EUS in 40%. Tumors were exclusively solitary (100%), with a median size of 1.0 cm (IQR: 0.8–1.0; range: 0.5–1.2), located primarily in the body of the pancreas (80%), with smaller locations in the head (10%) or tail (10%). There were no multifocal lesions in this group. Histological grading was G2 in 60%, G1 in 20%, and G3 in 20%. Metastases were metachronous in 90%, with a median time from nephrectomy of 107 months (IQR: 81–130; range: 3–190). Partial nephrectomy predominated in the treatment of the primary tumor (60%). Median blood loss was 150 mL (IQR: 100–200; range: 100–250), with no lymph nodes removed (median 0). Invasion was rare: lymphatic in 0%, vascular in 10%, and perineural in 10%. R0 margins were achieved in 100%. Clavien–Dindo complications predominated in grade 0 (90%), with no higher grades (III–V) or reoperations. Median hospitalization was 10.5 days (IQR: 10–11; range: 10–15). Median follow-up was 144 months (IQR: 105–190; range: 36–300). Local recurrences occurred in 10% and distant recurrences in 20%. Median PFS was 144 months (IQR: 94–190; range: 36–300), OS 144 months (IQR: 105–190; range: 36–300), with death occurring in 50%.

### 3.4. Analysis of Group B

Group B—patients undergoing resection (DP, PD, or TP)—included 50 patients, with a male predominance (65.4%). The median age was 64 years (IQR: 59–69; range: 48–74). A history of smoking was present in 53.8%, and the median BMI was 26.5 kg/m^2^ (IQR: 24.1–29.0; range: 20.5–33.6). ASA classification class II predominated (48.1%), with class III in 32.7% and class I in 19.2%. Preoperative diagnostic workup included CT in 94.2%, MRI in 75%, and EUS in 50%. Tumors were solitary in 71.2%, multiple in 28.8%, with a median size of 2.0 cm (IQR: 1.8–2.5; range: 1.0–5.0). Location of metastases: pancreatic head in 40.4%, body in 34.6%, tail in 48.1%, and multifocal in 19.2%. Histological grading was G2 in 57.7%, G1 in 23.1%, and G3 in 19.2%. Metastases were metachronous in 100%, with a median time from nephrectomy of 101.5 months (IQR: 73.5–141.5; range: 14–228). Treatment of the primary tumor was predominantly total nephrectomy (80.8%). Pancreatic procedures: DP in 59.6%, PD in 28.8%, TP in 11.5%. Median blood loss was 300 mL (IQR: 250–400; range: 150–600); number of nodes removed was 26 (IQR: 19–31.5; range: 14–66). Invasion: lymphatic in 25%, vascular in 40.4%, and perineural in 23.1%. Margins: R0 in 67.3%, R1 in 28.8%, none in 3.8%. Clavien–Dindo complications: grade 0 in 28.8%, I in 17.3%, II in 19.2%, III in 26.9%, IV in 1.9%, V in 5.8%; reoperations in 13.5%. Median hospital stay was 12.5 days (IQR: 11–19; range: 6–46). Median follow-up was 70 months (IQR: 36–120; range: 1–273). Local recurrence was 11.5%; distant recurrence was 30.8%. Median PFS was 61 months (IQR: 28–120; range: 0–273), and OS was 70 months (IQR: 33–120; range: 1–273), with death occurring in 53.8%.

### 3.5. Significant Differences Between Groups A and B

Subgroup comparisons revealed statistically significant differences in several aspects. Group A, compared to group B, had a higher prevalence of smoking history (100% vs. 53.8%; *p* = 0.005) and partial nephrectomy for primary tumor treatment (60% vs. 21.2%; *p* = 0.020), while total nephrectomy predominated in group B (40% vs. 80.8%; *p* = 0.014). Tumors in group A were smaller (median 1 cm vs. 2 cm; *p* < 0.001); exclusively single (100% vs. 71.2%; *p* = 0.100); and more frequently located in the body of the pancreas (80% vs. 34.6%; *p* = 0.013), less frequently in the tail (10% vs. 48.1%; *p* = 0.035) or head (*p* = 0.082). The primary tumor was located in the left kidney more frequently in group A (90% vs. 53.8%; *p* = 0.040). Resection procedures were associated with greater blood loss (median 300 mL vs. 150 mL; *p* < 0.001), a higher number of removed nodes (median 26 vs. 0; *p* < 0.001), and longer hospitalization (median 12.5 days vs. 10.5 days; *p* = 0.022). Clavien–Dindo complications were milder in group A, with grade 0 predominating (90% vs. 28.8%; *p* = 0.042 for the overall distribution). Follow-up was longer in group A (median 144 months vs. 70 months; *p* = 0.007), as were PFS (144 months vs. 61 months; *p* = 0.007) and OS (144 months vs. 70 months; *p* = 0.006). No significant differences were found in grading, invasion, recurrence, or death rates (*p* > 0.05).

### 3.6. OS and PFS Results After 1, 3, and 5 Years of Follow-Up in the Total Study Cohort (N = 62)

The estimates in Table 2, derived through PSW with tumor size incorporated as an additional covariate to address residual imbalances, indicate a favorable prognosis, with high OS rates: 95.9% at 1 year, 92.2% at 3 years, and 71.4% at 5 years. Progression-free survival (PFS) rates in Table 3 also demonstrate favorable long-term outcomes, particularly in the context of local recurrence, where the median PFS was 72 months (IQR: 36–144 months). The results for local recurrence indicate very high PFS rates: 100.0% at 1 year (event-free), 97.7% at 3 years, and 85.4% at 5 years. For distant metastases, PFS rates are slightly lower—99.4% at 1 year, 92.3% at 3 years, and 76.3% at 5 years—reflecting the higher risk of systemic progression compared with local recurrence.

### 3.7. Overall Survival (OS) Rates After 1, 3, and 5 Years of Follow-Up, According to Surgical Procedure

**Figure 2:**
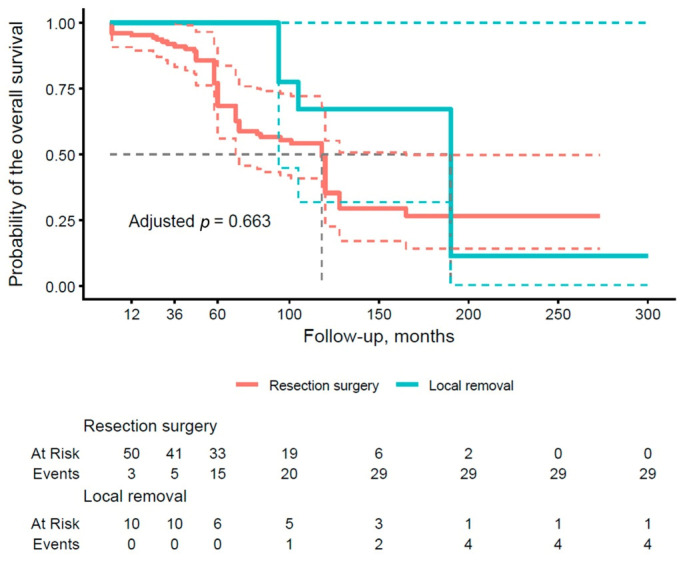
Kaplan–Meier curve of overall survival (OS) stratified by the type of surgical procedure (group A vs. group B). Thick, continuous lines represent the median 95% confidence interval (CI), and dashed lines illustrate the lower and upper limits of the 95% CI for each group.

The results presented in Table 4 illustrate the OS rates after 1, 3, and 5 years of follow-up, stratified by type of pancreatic surgery. With respect to local removal (group A), the surgical subgroup demonstrated an excellent OS of 100.0% (95% CI: 100–100) at all time points (1, 3, and 5 years), which contrasts with lower values in group B (89.5%, 83.2%, and 68.4%, respectively). The analysis shows a trend toward better OS rates in the subgroups with conservative approaches compared to more radical resections. Figure 2 illustrates the OS curve by type of pancreatic surgery (group A vs. group B). The curve for group B shows a more rapid decline in survival after 36 months, whereas group A maintains a more stable survival rate, remaining at 100% until after 60 months and approaching 60% at the end of the period. The adjusted *p*-value of 0.663 does not reach significance, implying no clear advantage of one approach over the other in terms of OS, although the trend reveals a potential benefit of sparing techniques in selected patients.

### 3.8. Local Recurrence (PFS) Rates at 1, 3, and 5 Years of Follow-Up, According to Surgical Procedure

**Figure 3:**
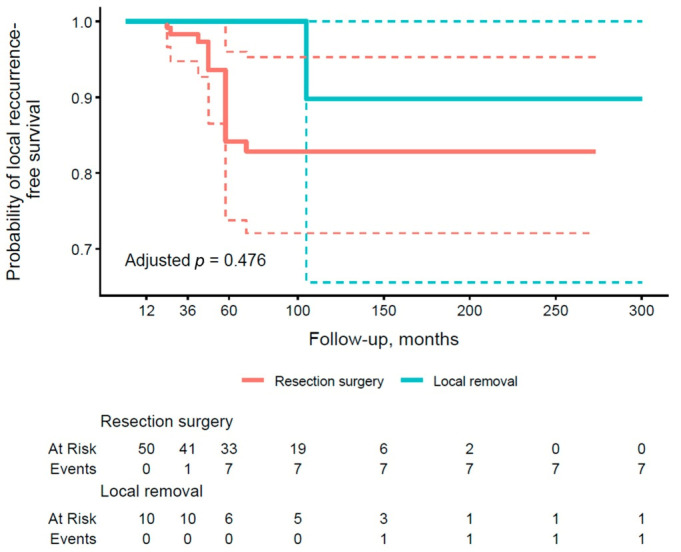
Kaplan–Meier curve of local recurrence-free survival stratified by the type of surgical procedure (group A vs. group B). Thick, continuous lines represent the median 95% confidence interval (CI), and dashed lines illustrate the lower and upper limits of the 95% CI for each group.

According to results in Table 5, group A again demonstrated excellent PFS of 100.0% at all time points, contrasting with lower rates in group B (100.0% at 1 year, 98.3% at 3 years, and 84.1% at 5 years). These results suggest a trend toward better PFS in the conservative treatment group for preventing local recurrence, consistent with patterns observed in OS rates. Figure 3 shows the PFS curve for local recurrence by type of operation. The curve for group B is lower, with more events accumulating by 60 months, while group A maintains a higher PFS throughout the period. The adjusted *p*-value of 0.476 does not reach significance, indicating no statistically significant effect of the type of procedure on this endpoint.

### 3.9. Distant Metastasis (PFS) Rates at 1, 3, and 5 Years of Follow-Up, Depending on the Type of Surgery Performed

**Figure 4:**
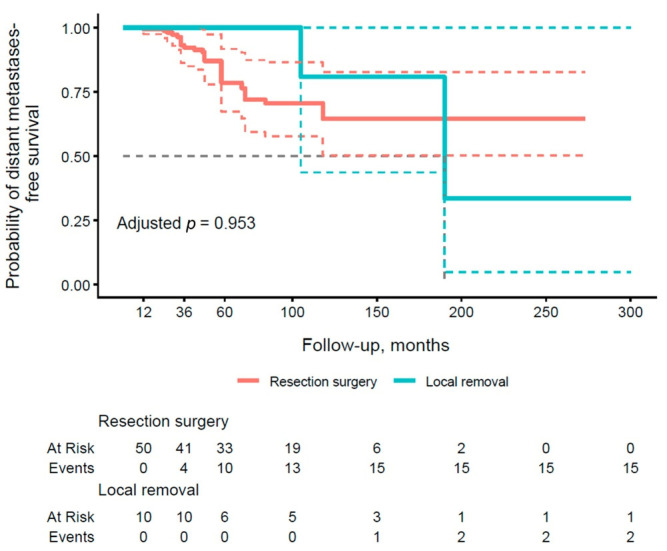
Kaplan–Meier curve of metastasis-free survival stratified by the type of surgical procedure (group A vs. group B). Thick, continuous lines represent the median 95% confidence interval (CI), and dashed lines illustrate the lower and upper limits of the 95% CI for each group.

The results presented in Table 6 illustrate distant metastasis PFS rates at 1, 3, and 5 years of follow-up, stratified by type of pancreatic surgery. With respect to local removal (group A), the subgroup demonstrated an excellent PFS of 100.0% (95% CI: 100.0–100.0) at all time points, contrasting with lower values in group B (99.4% at 1 year, 92.2% at 3 years, 78.6% at 5 years). Figure 4 illustrates the PFS curve for distant metastases by type of operation. The resection group (group B) demonstrated a lower PFS, with events accumulating by 60 months, but the adjusted *p*-value of 0.953 did not confirm significance.

### 3.10. Univariate and Multivariate Analysis of the Impact of Selected Demographic and Clinical Characteristics on OS

Univariate analysis in Table 7 highlights that the pathological features of the tumor, such as its size (HR 1.89; 95% CI: 1.24–2.90; *p* = 0.003), malignancy grade (grading G3 vs. G1: HR 2.95; 95% CI: 1.03–8.43; *p* = 0.043), lymphatic invasion (HR 9.15; 95% CI: 4.02–20.79; *p* < 0.001), vascular invasion (HR 6.13; 95% CI: 3.00–12.51; *p* < 0.001), and perineural invasion (HR 5.21; 95% CI: 2.53–10.73; *p* < 0.001), have an association with the prognosis of patients with metastatic renal cell carcinoma to the pancreas. R1 resection, as opposed to R0 (HR 10.68; 95% CI: 4.68–24.40; *p* < 0.001), highlights the importance of achieving tumor-free surgical margins. The symptoms of metastases as a proxy of clinical advancement (HR 3.85; 95% CI: 1.86–7.97; *p* < 0.001) have an association with a worse prognosis. Demographic factors such as gender (HR 1.34; 95% CI: 0.62–2.89; *p* = 0.460), age (HR 1.06; 95% CI: 0.99–1.13; *p* = 0.086), BMI (HR 1.01; 95% CI: 0.90–1.12; *p* = 0.922), smoking (HR 0.62; 95% CI: 0.31–1.23; *p* = 0.168), number of tumors (HR 0.66; 95% CI: 0.30–1.48; *p* = 0.317), multifocality of metastases (HR 1.94; 95% CI: 0.79–4.72; *p* = 0.146), and the type of pancreatic surgery (group A vs. group B: HR 2.05; 95% CI: 0.78–5.36; *p* = 0.143) did not reach statistical significance in this analysis.

In the multivariate analysis, after adjustment for demographic confounding variables (age, gender, and BMI), the model confirms the dominant independent role of pathological features and resection quality in predicting OS. Independent predictors associated with worse OS were larger tumor size (HR 1.97; 95% CI: 1.26–3.07; *p* = 0.003), lymphatic invasion (HR 8.87; 95% CI: 3.93–20.02; *p* < 0.001), vascular invasion (HR 6.67; 95% CI: 3.18–14.00; *p* < 0.001), perineural invasion (HR 7.27; 95% CI: 2.97–17.77; *p* < 0.001), R1 resection type (HR 11.34; 95% CI: 4.76–27.00; *p* < 0.001), and the presence of symptoms of metastases (HR 4.18; 95% CI: 1.95–8.98; *p* < 0.001).

### 3.11. Univariate and Multivariate Analysis of the Impact of Selected Demographic and Clinical Characteristics on Local Recurrence-Free Survival (PFS)

The univariate analysis presented in Table 8 indicates that lymphatic invasion (HR 22.73; 95% CI: 4.16–124.30; *p* < 0.001), vascular invasion (HR 9.28; 95% CI: 1.74–49.44; *p* = 0.009), and perineural invasion (HR 16.22; 95% CI: 3.0–85.41; *p* = 0.001) are associated with a significant increases in the risk of local recurrence. Similarly, the R1 resection (HR 36.35; 95% CI: 3.81–346.60; *p* = 0.002) and the symptoms of metastases (HR 7.16; 95% CI: 1.59–32.25; *p* = 0.010), was associated with a higher risk of progression. Demographic factors such as gender, age, and BMI, as well as smoking, tumor size, number of tumors, multifocality of metastases, grading, and type of pancreatic surgery (group A vs. group B), did not reach statistical significance.

In the multivariate analysis, after adjustment for age, gender and BMI, independent predictors associated with higher risk of local recurrence remained lymphatic invasion (HR 26.94; 95% CI: 4.19–173.47; *p* < 0.001), vascular invasion (HR 8.69; 95% CI: 1.55–48.84; *p* = 0.014), perineural invasion (HR 24.84; 95% CI: 3.03–203.87; *p* = 0.003), R1 resection type (HR 66.23; 95% CI: 4.10–1068.73; *p* = 0.003), and symptoms of the metastases (HR 8.00; 95% CI: 1.66–38.54; *p* = 0.010).

### 3.12. Univariate and Multivariate Analysis of the Impact of Selected Demographic and Clinical Characteristics on Distant Metastasis-Free Survival (PFS)

The univariate analysis in Table 9 shows that larger tumor size (HR 1.98; 95% CI: 1.14–3.44; *p* = 0.015) and G3 versus G1 staging (HR 6.20; 95% CI: 1.28–30.00; *p* = 0.023) increase the risk of distant metastases. Significant predictors also include lymphatic invasion (HR 13.51; 95% CI: 4.96–36.81; *p* < 0.001), vascular invasion (HR 34.24; 95% CI: 7.73–151.50; *p* < 0.001), perineural invasion (HR 14.18; 95% CI: 5.23–38.42; *p* < 0.001), R1 resection type (HR 21.15; 95% CI: 6.46–69.24; *p* < 0.001), and symptoms of the metastases (HR 7.25; 95% CI: 2.83–18.53; *p* < 0.001).

Demographic factors, smoking, number of tumors, multifocality of metastases, G2 grading, and type of pancreatic surgery did not reach significance. After adjustment for age, gender, and BMI in the multivariate analysis, independent risk factors associated with distant metastases remained tumor size (HR 2.03; 95% CI: 1.15–3.59; *p* = 0.014), G3 grading (HR 7.44; 95% CI: 1.31–42.40; *p* = 0.024), lymphatic invasion (HR 14.96; 95% CI: 5.24–42.75; *p* < 0.001), vascular invasion (HR 34.29; 95% CI: 7.46–157.73; *p* < 0.001), perineural invasion (HR 30.80; 95% CI: 7.79–121.77; *p* < 0.001), R1 resection type (HR 22.54; 95% CI: 6.53–77.89; *p* < 0.001), and symptoms of the metastases (HR 6.81; 95% CI: 2.62–17.67; *p* < 0.001). The remaining variables did not achieve significance, which indicates the central role of tumor histological features and resection margins.

### 3.13. Comparison of Characteristics of Metastases to the Pancreas According to the Location of the Primary Tumor

Comparison of metastasis location according to the side of the primary tumor in the kidneys (left vs. right) did not reveal any statistically significant differences (Table 10). For metastases to the head of the pancreas, the presence of lesions was noted in 25% of patients with tumors in the left kidney, 33.3% in the right kidney, and 37.5% in bilateral cases (*p* = 0.914), suggesting a lack of a preferential pattern of tumor migration associated with the primary side. Similarly, for metastases to the body of the pancreas, no lesions were found in any patients with tumors in the left kidney (100%), compared to 51.5% in the right kidney and 58.3% in bilateral cases (*p* = 0.211), which may indicate the random nature of the location, although the small size of the left kidney subgroup (N = 4) limits statistical power. In the case of metastases to the tail of the pancreas, the presence of lesions was dominant in the left kidney group (75%) and was lower in the right kidney group (42.4%) and the bilateral kidney group (37.5%; *p* = 0.422), which again did not reach the significance threshold.

## 4. Discussion

### 4.1. Characteristics of RCC Metastases to the Pancreas

RCC is the most common primary tumor that metastasizes to the pancreas [7,13,14]. RCC metastases to the pancreas are a rare manifestation of metastatic renal cell carcinoma (mRCC), characterized by the following features (Table 11) [15,16,17,18,19,20,21,22,23,24,25,26,27].

### 4.2. Late Recurrence

The tendency of RCC to produce late recurrences is a particularly remarkable feature. According to Noguchi G. et al. [16], the rate of RCC recurrence in retroperitoneal organs, including the contralateral kidney, pancreas, and adrenal gland, increased with time after surgery and showed the potential to recur even 10 years after nephrectomy. In the multicenter, retrospective PANMEKID study [17], RCC metastases to the pancreas appeared at a median of 87.35 months after primary surgery, and in the study by Malleo G. et al. [18], at 109 months. Andronelli et al. [19] reported the appearance of mRCC at 12.3 years, and Anderson B. et al. [12] at 8 years. In the study by Ma et al. [20], the median time between nephrectomy and surgery for mRCC to the pancreas was 11 years (range: 1–20 years), and in the Yuasa study [21], this median was as long as 13.4 years. In our study, the median occurrence of RCC metastases to the pancreas was 101.5 months after primary nephrectomy (IQR: 75–138, range: 3–2280), which is consistent with reports from the literature.

Late recurrence is, therefore, a specific biological behavior of RCC; however, the clinical and pathological features of late recurrence are not fully understood. In the study by Antonelli et al. [19], a total of 554 patients treated for RCC and followed for a median of 13.6 years were analyzed. RCC recurrence was observed in 26 patients (4.6%) after a median of 12.3 years. Pathological stage 2 or 3 was the only independent predictor of recurrence (*p* = 0.003) and was also associated with recurrence latency (median latency 14.0, 10.8, and 11.2 years for stages 1, 2, and 3, respectively; *p* < 0.005 for stage 1 versus stage 2 or 3). In the study by Miyao et al. [22], with a median follow-up of 13.2 years, 6.4% of patients experienced late relapse. Multivariate analysis showed that lymph node metastasis was the only predictor of late relapse (*p* = 0.0334), and age at nephrectomy was the only predictor of overall survival in multivariate analysis (*p* < 0.0001).

Considering the results of our study and the available data, despite the unknown mechanism of late recurrence, a 5-year follow-up period for RCC should be considered insufficient. Very long-term, or even lifelong, follow-up of patients with RCC should be considered.

### 4.3. Symptoms of mRCC to the Pancreas

RCC metastases to the pancreas are often asymptomatic and, therefore, go unnoticed and unreported by patients. They are often not detected during oncological surveillance for RCC but rather during routine imaging studies performed for other reasons [24,25]. In some studies, the authors report symptoms such as abdominal pain, nausea, gastrointestinal disturbances, pancreatitis, weight loss, jaundice, or gastrointestinal bleeding [24,25,26,27].

Our results confirm the nearly asymptomatic course of mRCC in the pancreas—79% of patients were asymptomatic. Symptoms of mRCC occurred in only 13 of 62 patients (21%), most commonly in the form of jaundice (6 of 13 patients), abdominal pain (3 of 13 patients), dyspeptic discomfort and symptoms (3 of 13 patients), and diarrhea (1 of 13 patients). Symptoms occurred with almost identical frequency in both study groups (group A—20%; group B—21.5%).

### 4.4. Good Prognosis

Numerous studies have highlighted the exceptionally good prognosis of patients with mRCC to the pancreas [16,17,18,19,20,21,22]. In Huang Q et al.’s [28] study of metastatic pancreatic tumors, patients with RCC as the primary tumor had significantly longer survival compared to patients with other diagnoses (*p* < 0.001). Similarly, in Lee et al.’s [29] study, OS and PFS were longer in patients with mRCC than in patients with other malignancies (*p* = 0.004 and *p* = 0.051, respectively). Roalsø MT [30] drew the same conclusions in a recent analysis: OS was best following pancreatectomy for mRCC metastases, approaching a median survival of 10 years.

Our study confirms the good prognosis after surgery for mRCC to the pancreas: the median OS is approximately 77 months, and 5-year survival reaches 71.4%.

### 4.5. Surgical Treatment of mRCC to the Pancreas

Surgery is a commonly used treatment method for mRCC to the pancreas. Based on 415 reported cases, cumulative 5-year and 10-year survival rates of 75.7% and 47.3%, respectively, were calculated [15]. According to Schwartz L. et al. [31], the 5-year survival rate for patients with untreated mRCC was 13% compared to 65% after surgical resection. In their retrospective study, Zerbi A. et al. [10] reported a survival rate of 47% in patients with mRCC treated nonoperatively compared to 88% in patients with surgical resection. In a series of 274 patients with mRCC to the pancreas (both metachronous and synchronous), Grassi et al. [32] found that patients treated surgically had twice the median OS compared to patients treated systemically with targeted therapies.

In our study, the overall survival rate for the entire cohort was 93.5% at 1 year, 84.9% at 3 years, and 75.3% at 5 years, with a median OS of 77.0 months, or approximately 6.4 years. These results emphasize that surgical procedures provide excellent outcomes in the treatment of pancreatic mRCC. A few cases of surgery performed due to synchronous disease were reported (up to approximately 7% [17])—in our study, only one patient (1.6%). Surgical treatment of these lesions is still debated. However, we believe that they should always be removed in conjunction with nephrectomy if the patient is in good health and the pancreas is the only organ involved [33].

### 4.6. Types of Surgical Procedures for Resection of Pancreatic RCC Metastases

There is no single, specifically recommended surgical procedure for resection of mRCC metastases to the pancreas. Surgical treatment typically involves standard pancreatic parenchymal resection, tailored to the tumor location, including pancreaticoduodenectomy, distal pancreatic resection, and total pancreatectomy [6]. Thanks to advances in surgical techniques, minimally invasive procedures can also be performed [34].

To plan the surgery, an abdominal CT with oral and intravenous contrast is needed to assess the size of the tumor and exclude infiltration of surrounding tissues. Additionally, intraoperative assessment of macroscopically unchanged pancreatic tissue is crucial. In our department, the resection is considered R0 if no tumor cells are found within 1 mm of the surgical incision line. R1 resection is diagnosed when tumor cells are found within 1 mm of the surgical incision line. These are very strict criteria, based on the NCCN guidelines for pancreatic adenocarcinoma, but in our unit, we commonly use them in pancreatic tumor surgery. Surgically, to reduce the risk of a positive R1 margin, we recommend maintaining at least a 1 mm margin of macroscopically unchanged pancreatic parenchyma around the tumor (in the case of local resection) and at least 1 mm of macroscopically unchanged pancreatic parenchyma around the margins typical for the type of resection (in the case of PD, around the superior mesenteric artery, superior mesenteric vein, anterior and posterior surfaces of the pancreas, and the incision margin in the isthmus of the pancreas; in the case of DP, around the anterior and posterior surfaces of the pancreas and the incision margin in the isthmus of the pancreas; in the case of TP, around the superior mesenteric artery, superior mesenteric vein, and anterior and posterior surfaces of the pancreas).

The issue of sparing surgery in the surgical treatment of mRCC to the pancreas remains unresolved. Isolated reports indicate favorable results with limited resection with preservation of the pancreatic parenchyma [35]. According to some authors, local resection (metastasectomy) is preferred over standard techniques (extensive resection) because oncological outcomes are comparable when only metastases are removed, and endocrine and exocrine function is preserved [36]. Zerbi et al. [10] also suggested that adequate local excision is not associated with a higher rate of recurrence compared with standard resection. However, Bassi et al. [37] report a high recurrence rate after limited resection, and they suggest that radical resection is necessary and that atypical local resection should be limited to certain exceptional cases.

In our study, only 10 patients (group A) underwent local resection, compared to 52 patients (group B) who underwent standard resection. Comparing group A with group B revealed several statistically significant differences. Analyzing the morphological characteristics of metastatic tumors, in group A, tumors were smaller (median 1 cm vs. 2 cm; *p* < 0.001) and were exclusively solitary (100% vs. 71.2%; *p* = 0.100, trend). Clavien–Dindo complications were milder in the local resection subgroup, with grade 0 predominating (90% vs. 28.8%; *p* = 0.042 for the overall distribution). In group B, blood loss was greater (median 300 mL vs. 150 mL; *p* < 0.001), and hospitalization was longer (median 12.5 days vs. 10.5 days; *p* = 0.022) compared to group A.

Surgical procedures performed on group B were, therefore, more invasive and burdensome for the patient, consistent with the greater extent of surgery in cases of pancreatic parenchymal resection compared to parenchymal-sparing tumor removal alone. The longer PFS (144 months vs. 61 months; *p* = 0.007) and OS (144 months vs. 70 months; *p* = 0.006) in group A compared with group B indicate a more favorable prognosis in group A, which is potentially related to the less invasive nature of the lesions and procedures. The analysis shows a trend toward better OS and PFS rates in the subgroups with parenchyma-conserving approaches compared with more radical resections. However, when we compared groups A and B, the log-rank test value did not reach significance for any of the endpoints studied (OS, PFS for local recurrence, and PFS for distant metastases). In multivariate analysis, the type of surgical treatment was not significantly associated with OS or PFS. However, given the numerous benefits of pancreatic parenchyma-sparing procedures (reduced risk of complications, death, and shorter hospital stay), we think that they should be used in cases of small, single metastases without signs of invasion (e.g., lymph node metastases or blood vessel invasion).

### 4.7. Factors Influencing OS

OS after surgical resection of RCC metastases to the pancreas is long—in the study by Boubaddi M et al. [38], the OS rate was as high as 92.8% after 5 years. According to our findings, the prognosis after surgical resection of RCC metastases to the pancreas is very good: median OS is approximately 77 months, and 5-year survival reaches 71.4%. In our multivariate analysis, after adjustment for demographic confounding variables (age, gender, and BMI), the independent predictors associated with worse OS were larger tumor size (HR 1.97; 95% CI: 1.26–3.07; *p* = 0.003), lymphatic invasion (HR 8.87; 95% CI: 3.93–20.02; *p* < 0.001), vascular invasion (HR 6.67; 95% CI: 3.18–14.00; *p* < 0.001), perineural invasion (HR 7.27; 95% CI: 2.97–17.77; *p* < 0.001), R1 resection type (HR 11.34; 95% CI: 4.76–27.00; *p* < 0.001), and the presence of metastases symptoms (HR 4.18; 95% CI: 1.95–8.98; *p* < 0.001). The loss of significance for G3 staging (HR 2.51; 95% CI: 0.82–7.71; *p* = 0.108) and multifocality of metastases (HR 2.33; 95% CI: 0.88–6.21; *p* = 0.089) after adjustment suggests that their effect may be partially mediated by demographic factors, such as older age or higher BMI, which themselves increase the risk of non-cancer death. These observations are partially consistent with published reports.

In Reddy S et al.’s [39] study of 21 patients who underwent pancreatic metastasectomy for RCC, tumor size greater than 4 cm (*p* = 0.13) and perineural invasion (*p* = 0.26) were not significantly associated with a difference in outcome, whereas lymph node involvement (HR 24.1; *p* = 0.01) and vascular invasion (HR 20.4; *p* = 0.007) were associated with poorer overall survival. Median survival was 4.8 years (range 0.35–18.3 years). Metachronic RCC had a survival similar to synchronous lesions (*p* = 0.98).

Kinoshita S et al. [40], in a multicenter study, enrolled 35 patients who underwent pancreatic resection due to metastases. The median follow-up period was 35 months (range: 5–102 months). The median time from resection of the primary tumor to resection of the metastatic pancreatic tumor was 10.6 years (range 0.6–29.2 years). The 3- and 5-year survival rates after resection of the metastatic pancreatic tumor were 89% and 69%, respectively. The 3- and 5-year disease-free survival rates after resection of the metastatic pancreatic tumor were 48% and 21%, respectively. Performance status ≥ 1 at the time of resection of the metastatic pancreatic tumor (HR: 7.56, *p* = 0.036) and metastatic pancreatic tumor diameter > 42 mm (HR: 6.39; *p* = 0.02) were significant prognostic factors for OS.

Resection margins seem to be crucial for improving OS in the surgical treatment of mRCC to the pancreas. In the study by Huang Q [27], a positive resection margin was associated with a worse prognosis (*p* = 0.035). Similarly, in the study by Chua et al. [41], the Cox proportional hazards model identified negative margin resection (HR 10.5; *p* = 0.044) as a predictor of improved survival. In our study, a positive margin (R1) was associated with worse OS (HR 11.34; 95% CI: 4.76–27.00; *p* < 0.001).

Numerous authors report that the presence of lymph node metastases is associated with a poor prognosis [31]. In the study by Milanetto AC [42], in the univariate analysis, lymph node metastases (HR 5.1; 95% CI 1.5–18), multiorgan resection (HR 3.4; 95% CI 1.1–10), and synchronous metastases of renal cell carcinoma (HR 13; 95% CI 3–55) were significantly associated with shorter OS. In our study, lymphatic invasion (HR 8.87; 95% CI: 3.93–20.02; *p* < 0.001) was also an independent predictor associated with worse OS.

In our study, vascular invasion (HR 6.67; 95% CI: 3.18–14.00; *p* < 0.001) was associated with worse OS. Similar observations were made by Tosojan JJ [43], where in a univariate analysis, vascular invasion (HR 5.15; *p* = 0.005) was significantly associated with an increased risk of death.

It should be emphasized, however, that factors influencing OS are not always identified. In a series of 20 patients with pancreatic metastases from RCC, Konstantinidis et al. [44] reported that in the univariate analysis, the number of metastatic nodules (single vs. multiple, *p* = 0.87), metastasis size (larger or smaller than a median of 3 cm, *p* = 0.78), metastasis location (*p* = 0.72), R1 resection (*p* = 0.62), and hemoglobin values below the lower reference range also did not predict worse outcomes (*p* = 0.99) and had no prognostic significance for overall survival.

A summary of histological factors that may influence prognosis can be found in Table 12.

### 4.8. Factors Influencing PFS

Recurrence of mRCC after resection is relatively frequently reported. In the study by Boubaddi M et al. [38], the PFS rate was 29.6% at 5 years, and in the study by Milanetto AC [42], the median PFS was 63 months, and 19 of 36 patients (52.77%) experienced disease recurrence.

In our study, in the multivariate analysis, independent predictors associated with a higher risk of local recurrence were lymphatic invasion (HR 26.94; 95% CI: 4.19–173.47; *p* < 0.001), vascular invasion (HR 8.69; 95% CI: 1.55–48.84; *p* = 0.014), perineural invasion (HR 24.84; 95% CI: 3.03–203.87; *p* = 0.003), R1 resection type (HR 66.23; 95% CI: 4.10–1068.73; *p* = 0.003), and the presence of symptoms of metastases (HR 8.00; 95% CI: 1.66–38.54; *p* = 0.010). The remaining factors did not retain significance, confirming the dominant role of invasive features and quality of resection in this model. The PFS profile for local recurrence is consistent with the OS results, with pathological features (invasion, R1 resection type, evidence of metastasis) also dominating as risk predictors, although G3 grading, which was univariately significant for OS, did not show an impact on PFS, and tumor size, which was significant in both univariate and multivariate analysis for OS, did not influence PFS.

In our patients, local recurrence was not treated surgically. However, it is worth noting that there are reports that emphasize prolonged OS with repeat pancreatic resection in cases of recurrence of RCC metastasis to the pancreatic remnant [45,46].

In the multivariate analysis, independent risk factors associated with distant metastases in our study were tumor size (HR 2.03; 95% CI: 1.15–3.59; *p* = 0.014), G3 staging (HR 7.44; 95% CI: 1.31–42.40; *p* = 0.024), lymphatic invasion (HR 14.96; 95% CI: 5.24–42.75; *p* < 0.001), vascular invasion (HR 34.29; 95% CI: 7.46–157.73; *p* < 0.001), perineural invasion (HR 30.80; 95% CI: 7.79–121.77; *p* < 0.001), R1 resection type (HR 22.54; 95% CI: 6.53–77.89; *p* < 0.001) and symptoms of the metastases (HR 6.81; 95% CI: 2.62–17.67; *p* < 0.001). The remaining variables did not retain significance. The PFS profile for distant metastases is, therefore, largely consistent with OS, with the dominance of pathological features and quality of resection, although G3 staging has a stronger effect on PFS than on OS, where it lost significance after adjustment, which may indicate differences in the mechanisms of systemic progression compared to OS.

### 4.9. Metastasis Pattern of RCC

In our study, the primary tumor (RCC) was more frequently located in the left kidney (59.7%) than in the right kidney (45.2%). However, the location of pancreatic metastases by the side of the primary tumor in the kidney (left vs. right) did not reveal any statistically significant differences. These observations indicate no correlation between the laterality of the primary tumor in the kidney and the specific location of pancreatic metastases.

This observation is confirmed by numerous reports. In their systematic review, Sellner et al. [15] emphasized that the correlation between the side of renal cell carcinoma and the site of metastasis within the pancreas was not statistically significant (N = 188; *p* = 0.876). Similarly, the study by Benhaim R. [10] found no correlation between the side of the primary renal tumor and the site of pancreatic metastases. The authors, therefore, conclude that lymphatic dissemination may not be the route of spread of mRCC metastases to the pancreas. Hematogenous dissemination also seems doubtful, as it does not explain the discrepancy between the occurrence of pancreatic metastases and the absence of metastases to other organs [6,10]. The most likely explanation for this unique behavior of isolated pancreatic metastases seems to be the high affinity of cancer cells for the pancreatic parenchyma [15]. This question certainly needs to be expanded upon in further research.

### 4.10. Study Limitations

Our study had some limitations. It was a retrospective cohort study with potential selection bias. Some patients were excluded from the study due to a lack of clinical data (N = 6). The number of patients in group A is significantly smaller than in group B (10 vs. 52 patients), which reduces the strength of the statistical analysis conclusions. Group A also had significantly different tumor features than group B, which, on the one hand, reduces the comparability of the groups, but on the other hand, shows that for small, solitary lesions, local resection may be chosen as beneficial. Patients were operated on by six different consultant surgeons during the study period, and the type of surgical procedure (local removal vs. resection surgery) was chosen by different people over many years, which may influence the results.

## 5. Conclusions

The prognosis after surgical resection of metastatic kidney cancer to the pancreas is excellent: median OS is approximately 77 months, and 5-year survival reaches 71.4%.

In the case of surgical treatment of metastatic renal cancer to the pancreas, in the multivariate analysis, the type of surgical treatment was not significantly associated with OS and PFS. However, pancreas-sparing procedures may be considered oncologically safe and beneficial in terms of recovery for well-defined, small (<1 cm), single metastases without obvious signs of invasion and without contact with the pancreatic duct. The decision on the type of surgery should be guided by preoperative CT results and the intraoperative assessment of the surrounding tissues.

The location of metastases in the pancreas does not show statistically significant differences considering the location of the primary tumor (left vs. right kidney).

## Figures and Tables

**Table 1:** Demographic, clinical, and pathological characteristics of the study cohort overall, divided into group A (local excision) and group B (resective surgery). Statistical tests used: Fisher’s exact test for small categorical variables, Wilcoxon’s rank-sum test for continuous variables, and Pearson’s chi-square test for sufficiently numerous categorical variables. *p*-values for the CD complication factor were performed using Holm’s correction for multiple complications. Statistically significant values (*p* < 0.05) are marked. Nomenclature used: Synchronous metastases—metastases detected at the same time or within a short time interval (<6 months) from the diagnosis of the primary tumor. Metachronous metastases—metastases detected within a significant time interval (>6 months) from the diagnosis of the primary tumor. Multiple lesions—the presence of multiple tumors (>1) located in the same area of the pancreas. Multifocal lesions—the presence of multiple tumors (>1) located in different areas of the pancreas. Local recurrence—tumor recurrence within the surgical site or regional lymph nodes. Distant metastases—disease recurrence elsewhere. Metastasis symptoms included jaundice (6 of 13 patients), abdominal pain (3 of 13 patients), dyspeptic discomfort (3 of 13 patients), and diarrhea (1 of 13 patients).

Characteristics	Total Cohort (N = 62)	Type of Surgical Procedure		*p*
		Group A Local Removal (N = 10)	Group B Resection Surgery (N = 52)	
I. Demographics				
Sex				
Women	21 (33.9%)	3 (30.0%)	18 (34.6%)	
Man	41 (66.1%)	7 (70.0%)	34 (65.4%)	
Age (years)	Median: 64 (IQR: 59–68.0) [range: 48–74]	Median: 64 (IQR: 59–68) [range: 48–71]	Median: 64.0 (IQR: 59–69) [range: 48–74]	0.694
Nicotine addiction	38 (61.3%)	10 (100%)	28 (53.8%)	**0.005**
BMI (kg/m^2^)	Median: 26.4 (IQR: 23.7–29) [range: 20.5–33.6]	Median: 25.5 (IQR: 23.7–30.4) [range: 21.8–31.9]	Median: 26.5 (IQR: 24.1–29) [range: 20.5–33.6]	0.759
II. Preoperative diagnostics				
TK	57 (91.9%)	8 (80%)	49 (94.2%)	0.180
MR	47 (75.8%)	8 (80%)	39 (75.0%)	1.000
EUS	30 (48.4%)	4 (40%)	26 (50%)	0.733
ASA				0.331
I	10 (16.1%)	0 (0%)	10 (19.2%)	
II	32 (51.6%)	7 (70%)	25 (48.1%)	
III	20 (32.3%)	3 (30%)	17 (32.7%)	
III. Data of metastatic tumor				
Size (cm)	Median: 2 (IQR: 1.4–2.5) [range: 0.5–5]	Median: 1 (IQR: 0.8–1) [range: 0.5–1.2]	Median: 2 (IQR: 1.8–2.5) [range: 1–5]	**<0.001**
Number of tumors				0.100
Multiple	15 (24.2%)	0 (0%)	15 (28.8%)	
Single	47 (75.8%)	10 (100%)	37 (71.2%)	
Location of the primary tumor (tumor may involve >1 localization)				
Left kidney	37 (59.7%)	9 (90%)	28 (53.8%)	**0.040**
Right kidney	28 (45.2%)	2 (20%)	26 (50.0%)	0.097
Location of the metastatic tumor				
Metastases to the head of the pancreas	22 (35.5%)	1 (10%)	21 (40.4%)	0.082
Metastases to the body of the pancreas	26 (41.9%)	8 (80%)	18 (34.6%)	**0.013**
Metastases to the tail of the pancreas	26 (41.9%)	1 (10%)	25 (48.1%)	**0.035**
Multifocal metastases	10 (16.1%)	0 (0%)	10 (19.2%)	0.194
Symptoms of the metastases	13 (21.0%)	2 (20%)	11 (21.5%)	1.000
Grading				
G1	14 (22.6%)	2 (20%)	12 (23.1%)	
G2	36 (58.1%)	6 (60%)	30 (57.7%)	
G3	12 (19.4%)	2 (20%)	10 (19.2%)	
Synchronous metastases to the pancreas	1 (1.6%)	1 (10%)	0 (0%)	0.161
Metachronous metastases to the pancreas	61 (98.4%)	9 (90%)	52 (100%)	0.161
IV. Treatment data				
Type of surgical procedure (kidney)				
Partial nephrectomy	17 (27.4%)	6 (60%)	11 (21.2%)	**0.020**
Total nephrectomy	46 (74.2%)	4 (40%)	42 (80.8%)	**0.014**
Type of surgical procedure (pancreas)				
Local removal	10 (16.1%)	10 (100%)	0 (0%)	**<0.001**
Distal pancreatectomy (DP)	31 (50%)	0 (0%)	31 (59.6%)	**0.001**
Pancreaticoduodenectomy (PD)	15 (24.2%)	0 (0%)	15 (28.8%)	0.100
Total pancreatectomy (TP)	6 (9.7%)	0 (0%)	6 (11.5%)	0.577
Blood loss (mL)	Median: 300 (IQR: 200–400) [range: 100–600]	Median: 150 (IQR: 100–200) [range: 100–250]	Median: 300 (IQR: 250–400) [range: 150–600]	**<0.001**
Total number of lymph nodes removed	Median: 24.5 (IQR: 17–29) [range: 0–66]	Median: 0 (IQR: 0–0) [range: 0–0]	Median: 26 (IQR: 19–31.5) [range: 14–66]	**<0.001**
Lymphatic invasion	13 (21%)	0 (0%)	13 (25%)	0.103
Vascular invasion	22 (35.5%)	1 (10%)	21 (40.4%)	0.082
Perineural invasion	13 (21%)	1 (10%)	12 (23.1%)	0.673
Type of resection				0.088
Lack of information	2 (3.2%)	0 (0.0%)	2 (3.8%)	
R0	45 (72.6%)	10 (100.0%)	35 (67.3%)	
R1	15 (24.2%)	0 (0.0%)	15 (28.8%)	
Time between nephrectomy and the appearance of pancreatic metastases (months)	Median: 101.5 (IQR: 75–138) [range: 3–228]	Median: 107 (IQR: 81–130) [range: 3–190]	Median: 101.5 (IQR: 73.5–141.5) [range: 14–228]	0.752
Complications according to Clavien–Dindo				
0	24 (38.7%)	9 (90%)	15 (28.8%)	**0.042**
I	9 (14.5%)	0 (0%)	9 (17.3%)	0.740
II	11 (17.7%)	1 (10%)	10 (19.2%)	1.000
III	14 (22.6%)	0 (0%)	14 (26.9%)	0.260
IV	1 (1.6%)	0 (0%)	1 (1.9%)	1.000
V	3 (4.8%)	0 (0%)	3 (5.8%)	1.000
Re-operation	7 (11.3%)	0 (0%)	7 (13.5%)	0.586
Hospitalization time (days)	Median: 12 (IQR: 10–18) [range: 6–46]	Median: 10.5 (IQR: 10–11) [range: 10–15]	Median: 12.5 (IQR: 11–19) [range: 6–46]	**0.022**
Observation time (months)	Median: 77 (IQR: 40–140) [range: 1–300]	Median: 144 (IQR: 105–190) [range: 36–300]	Median: 70 (IQR: 36–120) [range: 1–273]	**0.007**
V. Recurrence				
Progression for local recurrence	7 (11.3%)	1 (10%)	6 (11.5%)	1.000
Progression for distant metastases	18 (29.0%)	2 (20%)	16 (30.8%)	0.709
VI. Endpoints				
Progression-free survival (months)	Median: 72 (IQR: 36–144) [range: 0–300]	Median: 144 (IQR: 94–190) [range: 36–300]	Median: 61 (IQR: 28–120) [range: 0–273]	**0.007**
Overall survival (months)	Median: 77 (IQR: 36–144) [range: 1–300]	Median: 144 (IQR: 105–190) [range: 36–300]	Median: 70 (IQR: 33–120) [range: 1–273]	**0.006**
Death	33 (53.2%)	5 (50%)	28 (53.8%)	1.000

**Table 2:** OS rates after 1, 3, and 5 years of follow-up in the entire study cohort.

Observation Time (Years)	OS Rate (%)	95% Confidence Interval (CI)
1	95.9	91.1–1.00
3	92.2	85.6–99.3
5	71.4	60.0–85.0

**Table 3:** PFS rates after 1, 3, and 5 years of follow-up for local recurrence and distant metastases in the entire study cohort.

Observation Time (Years)	Progression for Distant Metastases		Progression for Local Recurrence	
	95% CI	PFS Rate (%)	95% CI	PFS Rate (%)
1	97.5–100.0	99.4	100.0–100.0	100.0
3	85.6–99.6	92.3	93.8–100.0	97.7
5	65.4–89.0	76.3	76.0–96.0	85.4

**Table 4:** OS rates after 1, 3, and 5 years of follow-up in groups A and B according to the type of surgery.

Operation	Group	Follow-Up (Years)	Number at Risk	Number of Events	OS (%)	95% CI
Type of surgical procedure	Resection Surgery Group B	1	50	3	89.5	89.5–100.0
		3	41	5	83.2	83.2–99.6
		5	33	15	68.4	56.1–83.6
	Local Removal Group A	1	10	0	100.0	100.0–100.0
		3	10	0	100.0	100.0–100.0
		5	6	0	100.0	100.0–100.0

**Table 5:** PFS rates of local recurrence after 1, 3, and 5 years of follow-up in groups A and B.

Operation	Group	Follow-Up (Years)	Number at Risk	Number of Events	OS (%)	95% CI
Type of surgical procedure	Resection Surgery Group B	1	50	0	100.0	100.0–100.0
		3	41	1	98.3	94.7–100.0
		5	33	7	84.1	73.8–96.0
	Local Removal Group A	1	10	0	100.0	100.0–100.0
		3	10	0	100.0	100.0–100.0
		5	6	0	100.0	100.0–100.0

**Table 6:** PFS rates of distant metastases after 1, 3, and 5 years of follow-up in groups A and B according to the type of surgery.

Operation	Group	Follow-Up (Years)	Number at Risk	Number of Events	OS (%)	95% CI
Type of surgical procedure	Resection Surgery Group B	1	50	0	99.4	97.4–100.0
		3	41	4	92.2	84.9–100.0
		5	33	10	78.6	67.3–91.7
	Local Removal (Group A)	1	10	0	100.0	100.0–100.0
		3	10	0	100.0	100.0–100.0
		5	6	0	100.0	100.0–100.0

**Table 7:** Univariate and multivariate analyses using Cox regression models of the effect of selected demographic and clinical characteristics on overall survival (OS). Statistically significant values (*p* < 0.05) are marked. HR (hazard ratio) values > 1 indicate an increased risk of death. Multivariate analysis adjusts for the effects of age, sex, and BMI.

Parameter	Univariate Analysis			Multivariate Analysis		
	HR	95% CI	*p*	HR	95% CI	*p*
Sex (male vs. female)	1.34	0.62–2.89	0.460	–	–	–
Age	1.06	0.99–1.13	0.086	–	–	–
BMI	1.01	0.90–1.12	0.922	–	–	–
Smoking	0.62	0.31–1.23	0.168	0.59	0.30–1.20	0.146
Tumor size (cm)	1.89	1.24–2.90	**0.003**	1.97	1.26–3.07	**0.003**
Number of tumors (single vs. multiple)	0.66	0.30–1.48	0.317	0.55	0.23–1.29	0.166
Multifocal vs. single-focal meta	1.94	0.79–4.72	0.146	2.33	0.88–6.21	0.089
Grading 2 vs. Grading 1	1.45	0.57–3.69	0.439	1.16	0.45–3.04	0.755
Grading 3 vs. Grading 1	2.95	1.03–8.43	**0.043**	2.51	0.82–7.71	0.108
Type of surgical procedure (resection vs. removal)	2.05	0.78–5.36	0.143	1.93	0.73–5.08	0.183
Lymphatic invasion (yes vs. no)	9.15	4.02–20.79	**<0.001**	8.87	3.93–20.02	**<0.001**
Vascular invasion (yes vs. no)	6.13	3.00–12.51	**<0.001**	6.67	3.18–14.00	**<0.001**
Perineural invasion (yes vs. no)	5.21	2.53–10.73	**<0.001**	7.27	2.97–17.77	**<0.001**
Type of resection (R1 vs. R0)	10.68	4.68–24.40	**<0.001**	11.34	4.76–27.00	**<0.001**
Symptoms of the metastases (yes vs. no)	3.85	1.86–7.97	**<0.001**	4.18	1.95–8.98	**<0.001**

**Table 8:** Univariate and multivariate analyses using Cox regression models of the effect of selected demographic and clinical characteristics on local recurrence-free survival (PFS). Statistically significant values (*p* < 0.05) are marked. HR (hazard ratio) values >1 indicate an increased risk of death. Multivariate analysis adjusts for the effects of age, sex, and BMI.

Parameter	Univariate Analysis			Multivariate Analysis		
	HR	95% CI	*p*	HR	95% CI	*p*
Sex (male vs. female)	0.59	0.13–2.64	0.489	–	–	–
Age	1.00	0.88–1.14	0.995	–	–	–
BMI	0.84	0.62–1.09	0.184	–	–	–
Smoking	0.37	0.08–1.66	0.194	0.42	0.09–1.96	0.268
Tumor size (cm)	0.94	0.33–2.65	0.900	0.99	0.36–2.75	0.984
Number of tumors (single vs. multiple)	0.56	0.11–2.92	0.497	0.77	0.13–4.48	0.773
Multifocal vs. single-focal meta	1.32	0.16–11.04	0.798	0.79	0.08–7.85	0.842
Grade 2 vs. grade 1	0.70	0.12–4.17	0.691	1.02	0.14–7.26	0.984
Grade 3 vs. grade 1	1.73	0.24–12.33	0.587	1.59	0.20–12.81	0.662
Type of surgical procedure (resection vs. removal)	1.67	0.20–14.01	0.636	1.82	0.20–16.47	0.594
Lymphatic invasion (yes vs. no)	22.73	4.16–124.30	**<0.001**	26.94	4.19–173.47	**<0.001**
Vascular invasion (yes vs. no)	9.28	1.74–49.44	**0.009**	8.69	1.55–48.84	**0.014**
Perineural invasion (yes vs. no)	16.22	3.0–85.41	**0.001**	24.84	3.03–203.87	**0.003**
Type of resection (R1 vs. R0)	36.35	3.81–346.60	**0.002**	66.23	4.10–1068.73	**0.003**
Symptoms of the metastases (yes vs. no)	7.16	1.59–32.25	**0.010**	8.00	1.66–38.54	**0.010**

**Table 9:** Univariate and multivariate analyses using Cox regression models of the effect of selected demographic and clinical characteristics on distant metastasis-free survival (PFS). Statistically significant values (*p* < 0.05) are marked. Hazard ratio (HR) values >1 indicate an increased risk of death. Multivariate analysis adjusts for the effects of age, sex, and BMI.

Parameter	Univariate Analysis			Multivariate Analysis		
	HR	95% CI	*p*	HR	95% CI	*p*
Sex (male vs. female)	0.48	0.19–1.22	0.122	–	–	–
Age	1.00	0.93–1.08	0.946	–	–	–
BMI	0.92	0.80–1.07	0.277	–	–	–
Smoking	0.40	0.16–1.03	0.057	0.46	0.18–1.21	0.116
Tumor size (cm)	1.98	1.14–3.44	**0.015**	2.03	1.15–3.59	**0.014**
Number of tumors (single vs. multiple)	0.58	0.20–1.63	0.300	0.65	0.22–1.90	0.429
Multifocal vs. single-focal meta	2.31	0.76–7.07	0.141	2.15	0.64–7.25	0.218
Grade 2 vs. grade 1	2.06	0.44–9.57	0.356	2.82	0.53–15.04	0.224
Grade 3 vs. grade 1	6.20	1.28–30.00	**0.023**	7.44	1.31–42.40	**0.024**
Type of surgical procedure (resection vs. removal)	2.41	0.55–10.59	0.243	2.46	0.55–11.09	0.240
Lymphatic invasion (yes vs. no)	13.51	4.96–36.81	**<0.001**	14.96	5.24–42.75	**<0.001**
Vascular invasion (yes vs. no)	34.24	7.73–151.50	**<0.001**	34.29	7.46–157.73	**<0.001**
Perineural invasion (yes vs. no)	14.18	5.23–38.42	**<0.001**	30.80	7.79–121.77	**<0.001**
Type of resection (R1 vs. R0)	21.15	6.46–69.24	**<0.001**	22.54	6.53–77.89	**<0.001**
Symptoms of the metastases (yes vs. no)	7.25	2.83–18.53	**<0.001**	6.81	2.62–17.67	**<0.001**

**Table 10:** Comparison of the location of pancreatic metastases depending on the side of the primary tumor in the kidney (left vs. right) in 61 * patients who underwent surgical treatment of renal cell carcinoma metastases to the pancreas.

Location of Pancreatic Metastases	Presence of Metastasis	Left Kidney (N = 4)	Right Kidney (N = 33)	Involvement of Both Kidneys (N = 24)	*p*
Metastases to the head of the pancreas	No	3 (75%)	22 (66.7%)	15 (62.5%)	0.914
	Yes	1 (25%)	11 (33.3%)	9 (37.5%)	
Metastases to the body of the pancreas	No	4 (100%)	17 (51.5%)	14 (58.3%)	0.211
	Yes	0 (0%)	16 (48.5%)	10 (41.7%)	
Metastases to the tail of the pancreas	No	1 (25%)	19 (57.6%)	15 (62.5%)	0.422
	Yes	3 (75%)	14 (42.4%)	9 (3.5%)	

* 1 patient excluded from the analysis—no data.

**Table 11:** Characteristic features of pancreatic mRCC.

Feature	Pancreatic mRCC
Type of metastasis	• Most often, metastases occur exclusively in the pancreas, rarely with synchronous metastases. - In the literature: 0–29% [15]. - In our study: 1.6%.
Recurrence	• The appearance of pancreatic metastases after a long period of follow-up following primary RCC treatment (“late recurrence”). - In the literature: median 7.25–13.4 years [16,17,18,19,20,21,22]. - In our study: median approximately 8.5 years.
Prognosis	• Unusually low tumor aggressiveness with slow tumor progression and good treatment outcomes. - In the literature, median OS up to 10 years [16,17,18,19,20,21,22,28,29,30]. - In our study, median OS is approximately 6.4 years.
Histological structure	• Uniform histological structure with low-grade malignant tumor cells and a highly vascular stroma devoid of inflammatory cells [23].
Symptoms	• The course is usually asymptomatic. - In the literature, up to 93% of patients are asymptomatic [24,25,26,27]. - In our study, 79% of patients are asymptomatic.

**Table 12:** Histological features and their impact on prognosis in mRCC according to the literature. NR—not reported.

Histological Feature	Yes (*p* < 0.05)	No (*p* > 0.05)
Tumor size	• Worse OS: metastatic tumor diameter > 42 mm; *p* = 0.02 [40]	• No impact on OS: metastatic pancreatic tumor diameter > 40 mm (*p* = 0.13) [39] No impact on OS: metastasis size (larger or smaller than a median of 3 cm, *p* = 0.78) [44]
Number of metastatic lesions	• NR	• No impact on OS: single vs. multiple, *p* = 0.87 [44]
Perineural invasion	• NR	• No impact on OS: *p* = 0.26 [39]
Lymphatic invasion	• Worse OS [31, 42—HR 5.1; 95% CI 1.5–18]	• No impact on OS: HR 24.1; *p* = 0.01 [39]
Vascular invasion	• NR	• No impact on OS: HR 20.4; *p* = 0.007 [39]
Positive resection margin	• Worse OS (*p* = 0.035) [27]	• No impact on OS: R1 resection (*p* = 0.62) [44]
Negative resection margin	• Predictor of improved OS; *p* = 0.044) [41]	• NR
Synchronous metastases	• Worse OS: HR 13; 95% CI 3–55 [42]	• No impact: *p* = 0.98 [39]

## Data Availability

The data are available from the corresponding author upon reasonable request.

## Supplementary Materials

The following supporting information can be downloaded at: https://www.mdpi.com/article/10.3390/cancers18010004/s1, Table S1. List of covariates incorporated into the propensity score model, including their type, observed imbalances, and rationale for inclusion based on statistical and clinical criteria. Table S2. Key covariates omitted from the PSW, with justifications centered on balance status, prognostic relevance, and methodological constraints to prevent overfitting. Table S3. Primary limitations associated with the PSM approach, emphasizing constraints imposed by sample size and data characteristics. Table S4. Distribution of Stabilized Weights and Extreme Values by Treatment Group in the Propensity Score Weighting Model. Table S5. Descriptive Statistics of Stabilized Weights by Treatment Group. Table S6. Effective Sample Sizes. Table S7. Unadjusted and Adjusted Standardized Mean Differences for Covariates Before and After Propensity Score Weighting.

## Abbreviations

The following abbreviations are used in this manuscript:

ASA	American Society of Anesthesiologists
BMI	Body Mass Index
ccRCC	Clear Cell Renal Cell Carcinoma
CI	Confidence Interval
CT	Computed Tomography
DP	Distal Pancreatectomy
EUS	Endoscopic Ultrasonography
HR	Hazard Ratio
IQR	Interquartile Range
Mdn	Median
MR	Magnetic Resonance
mRCC	Metastatic Renal Clear Carcinoma
NR	Not Reported
OS	Overall Survival
PD	Pancreaticoduodenectomy
PFS	Progression Free Survival
PSW	Propensity Score Weighting
RCC	Renal Clear Carcinoma
TP	Total Pancreatectomy
