# Supplementary material for: Surgical Treatment Options for Renal Cell Carcinoma Metastases to the Pancreas—25 Years of Single-Center Experience
Source: Cancers (Basel). 2025 Dec 19;18(1):4. doi: 10.3390/cancers18010004 (PMC12785051; doi:10.3390/cancers18010004)
Supplement: Supplementary file 1 [file cancers-18-00004-s001.zip › cancers-4033917-supplementary.pdf]

Propensity Score Matching for Covariate Balancing

To mitigate selection bias and confounding in the retrospective comparison between local tumor removal (Group A) and classical pancreatic resection (Group B), propensity score weighting (PSW) was applied, targeting the average treatment effect (ATE) estimand. This approach assigns weights to observations such that the weighted distributions of baseline covariates are balanced across groups, facilitating unbiased estimation of treatment effects. The propensity scores were initially derived from a model incorporating five covariates: tumor size (continuous, in cm), smoking history (presence of nicotine addiction; binary), type of nephrectomy for the primary tumor (total versus partial; binary), number of lesions (single versus multiple; binary), and primary tumor location in the left kidney (yes versus no; binary). These covariates were selected based on observed imbalances and their potential to confound surgical allocation and oncological outcomes, as identified through preliminary univariate comparisons (see Tables S1–S3). Specifically, Table S1 provides a detailed justification for the list of covariates incorporated into the PSW model, outlining their types, evidence of imbalance, and clinical rationale. Table S2 enumerates the covariates omitted from the model, with explanations centered on minimal imbalances, lack of prognostic relevance, or methodological constraints to prevent overfitting. Additionally, Table S3 summarizes the primary limitations associated with the PSW approach, emphasizing constraints imposed by the sample size and data characteristics, such as risks of residual confounding and reduced effective sample sizes post-weighting.

Table S1. List of covariates incorporated into the propensity score model, including their type, observed imbalances, and rationale for inclusion based on statistical and clinical criteria.

Covariate	Type	Imbalance Evidence	Rationale for Inclusion
Tumor size (cm)	Continuous	Median: 1.0 cm (Group A) vs. 2.0 cm (Group B); p < 0.001	Prioritized as a key morphological confounder; independently associated with worse OS (HR 1.97; 95% CI: 1.26–3.07; p=0.003) and distant PFS (HR 2.03; 95% CI: 1.15–3.59; p=0.014).
Smoking history	Binary (yes/no)	Prevalence: 100% (Group A) vs. 53.8% (Group B); p=0.005	Reflects baseline lifestyle factor potentially influencing perioperative risk and prognosis.
Type of nephrectomy (primary tumor)	Binary (partial vs. total)	Partial: 60% (Group A) vs. 21.2% (Group B), p=0.020; Total: 40% (Group A) vs. 80.8% (Group B), p=0.014	Serves as proxy for initial disease extent and surgical history, confounding metastatic management.
Primary tumor location (left	Binary (yes/no)	Prevalence: 90% (Group A) vs. 53.8% (Group B);	Captures anatomical asymmetries explored in the study, though without

Covariate	Type	Imbalance Evidence	Rationale for Inclusion
kidney)		p=0.040	direct prognostic impact.
Number of lesions	Binary (single vs. multiple)	Prevalence: 100% single (Group A) vs. 71.2% (Group B); p=0.100 (trend)	Aligns with multifocality trends; influences recurrence risk as a baseline imaging attribute.

Table S2. Key covariates omitted from the PSW, with justifications centered on balance status, prognostic relevance, and methodological constraints to prevent overfitting.

Excluded Covariate	Type/Category	Reason for Exclusion
Age	Continuous	Negligible imbalance (p > 0.05, low SMD); no evidence of confounding survival endpoints.
Sex	Binary	Negligible imbalance (p > 0.05, low SMD); lacks confounding potential.
Body mass index (BMI)	Continuous	Negligible imbalance (p > 0.05, low SMD); not associated with outcomes.
ASA classification	Categorical	Negligible imbalance (p > 0.05, low SMD); irrelevant to baseline confounding.
Histological grading (G1/G2/G3)	Categorical	Comparable distributions (p > 0.05); prognostic role variable and diminished post-adjustment.
Time from nephrectomy	Continuous	Balanced (p > 0.05); no baseline confounding.
Presence of symptoms	Binary	Balanced (20% vs. 21.5%; p > 0.05); prognostic but not imbalanced.
Imaging modalities (CT/MRI/EUS)	Categorical	Balanced (p > 0.05); absence of prognostic or confounding role.
Postoperative features (e.g., invasions, margins)	Pathological	Ascertained post-treatment; inappropriate for PSM due to risk of reverse causation bias.

Table S3. Primary limitations associated with the PSM approach, emphasizing constraints imposed by sample size and data characteristics.

Limitation Category	Description
Sample Size Constraints	Modest cohort (N=62; Group A n=10) limits model to 3–5 covariates per empirical guideline (1 per 8–10 minority group events), risking residual confounding.
Risk of Overfitting/Instability	Inclusion of near-perfect predictors (e.g., 100% single lesions in Group A) may cause quasi-separation; requires remedies like penalized likelihood, potentially compromising balance.
Reduced Effective Sample	1:1 matching with caliper (0.2 SD) may yield ≤10 pairs, attenuating power and generalizability; alternative weighting considered but not adopted.
Unmeasured Confounders	Retrospective data precludes adjustment for unrecorded variables; exploratory analysis may not fully eliminate bias.
Post-Matching Assessment	Balance evaluated via SMD (<0.10) and variance ratios, but small samples may inflate type I/II errors in subsequent survival analyses.

Weights were estimated using energy balancing, a method that minimizes the energy distance between the weighted covariate distributions of the two groups while stabilizing the weights to reduce variance and prevent extreme values from unduly influencing results. Stabilization ensures that weights are bounded and centered around unity, enhancing the robustness of subsequent analyses. The procedure aimed to achieve covariate balance by iteratively adjusting weights until the standardized mean differences (SMDs) were minimized. However, despite these efforts, tumor size could not be adequately balanced due to its continuous nature and the limited sample size, which constrained overlap and led to persistent imbalances post-weighting.

Consequently, the final weighting model proceeded with the four binary covariates (smoking history, type of nephrectomy, number of lesions, and primary tumor location), achieving acceptable balance for these parameters. Covariate balance was assessed using absolute SMDs, computed with a pooled standard deviation to accommodate potential zero-variance scenarios in binary variables. An SMD threshold of 0.2 was employed to determine acceptable balance, representing a moderate criterion that balances stringency with feasibility in small samples, where stricter thresholds (e.g., 0.1) may lead to excessive sample inefficiency without substantial gains in bias reduction. Effective sample sizes were calculated post-weighting to evaluate the impact on statistical efficiency.

This weighted framework was then utilized for Kaplan-Meier survival analyses of overall survival and progression-free survival. To account for the unresolved imbalance in tumor size, which was identified as a significant prognostic factor in multivariate analyses, it was incorporated as a covariate in the survival models through stratification in the Kaplan-Meier estimation and inclusion in subsequent Cox proportional hazards regression, ensuring adjustment for its confounding influence. Interpretations remain cautious of potential residual confounding due to unmeasured variables inherent in retrospective data.

The propensity score weighting model was successfully implemented to balance baseline covariates between the treatment groups, yielding stabilized weights that minimize selection bias. The following sections detail the weight distribution, statistical characteristics, and covariate balance assessment, with interpretations provided for each component.

The weights exhibited moderate variability, reflecting the adjustments necessary to achieve covariate balance in a small sample. Table S.4 summarizes the weight ranges and the five most extreme weights by group.

Table S4. Distribution of Stabilized Weights and Extreme Values by Treatment Group in the Propensity Score Weighting Model

Aspect	Treated Group (n=52 unweighted)	Control Group (n=10 unweighted)

Aspect	Treated Group (n=52 unweighted)	Control Group (n=10 unweighted)
Minimum Weight	0.419	0.501
Maximum Weight	3.864	3.713
Extreme Weights (Top 5)	3.864 (Obs 2, 3, 13), 1.643 (Obs 14, 25)	3.713 (Obs 4), 1.094 (Obs 7, 8, 9), 0.501 (Obs 10)

Notes: Weights were stabilized to mitigate variance inflation, with values centered around unity. The treated group corresponds to classical pancreatic resection (n=52 unweighted), and the control group to local tumor removal (n=10 unweighted). Extreme weights are listed by observation identifiers for transparency.

No weights exceeded four times the median, indicating controlled heterogeneity. The presence of weights below 1 in both groups implies downweighting of units with poor overlap, which is appropriate for enhancing comparability but may contribute to effective sample size reductions.

Descriptive statistics for the weights are presented in Table S.5, highlighting measures of dispersion and distribution.

Table S5. Descriptive Statistics of Stabilized Weights by Treatment Group

Group	Coefficient of Variation	Mean Absolute Deviation	Entropy	Number of Zero Weights
Treated	0.900	0.715	0.317	0
Control	0.993	0.599	0.309	0

The coefficients of variation near 1.0 denote substantial but controlled heterogeneity in weights, with entropy values indicating a moderate spread without excessive concentration. This profile supports the model's efficiency in reweighting without introducing instability, as zero weights were absent, ensuring all observations contributed to the analysis albeit differentially.

Post-weighting effective sample sizes, which account for the information loss due to unequal weighting, are outlined in Table S.6.

Table S6. Effective Sample Sizes

Sample Type	Control	Treated
Unweighted	10	52
Weighted	5.3	28.98

The reductions (approximately 47% for control and 44% for treated) reflect the trade-off between balance and precision, common in propensity score applications with imbalanced group sizes. While this diminishes statistical power, it enhances the validity of causal inferences by prioritizing covariate equivalence.

Table S.7 presents the unadjusted and adjusted SMDs for each covariate.

Table S7. Unadjusted and Adjusted Standardized Mean Differences for Covariates Before and After Propensity Score Weighting

**Covariate**	**Type**	**Unadjusted SMD**	**Adjusted SMD**	**Threshold Status**
Nicotine Addiction (Yes)	Binary	-0.4615	-0.1935	Balanced (<0.2)
Type of Nephrectomy (Total)	Binary	0.3885	0.0132	Balanced (<0.2)
Number of Lesions (Single)	Binary	-0.2885	-0.1210	Balanced (<0.2)
Primary Tumor Location (Left Kidney, Yes)	Binary	-0.3615	-0.0160	Balanced (<0.2)

All covariates achieved balance post-weighting, with the largest residual SMD (0.1935 for nicotine addiction) falling well below the threshold. This improvement from unadjusted values demonstrates the weighting's effectiveness in mitigating confounding, though interpretations should consider the binary nature of variables, which may limit sensitivity to subtle imbalances.The balance tally confirmed no covariates exceeded the threshold (4 balanced, 0 imbalanced), and nicotine addiction exhibited the greatest adjusted difference, underscoring its partial but acceptable resolution.

Overall, these results affirm the propensity score weighting model's utility in producing comparable groups, facilitating robust downstream survival analyses while acknowledging sample size constraints.
